# Machine Learning for Personalized Prediction of Electrocardiogram (EKG) Use in Emergency Care

**DOI:** 10.3390/jpm15080358

**Published:** 2025-08-06

**Authors:** Hairong Wang, Xingyu Zhang

**Affiliations:** 1 Department of Civil and Environmental Engineering, Carnegie Mellon University, Pittsburgh, PA 15213, USA; hairongw@andrew.cmu.edu; 2 Master of Science in Analytics Program, Georgia Institute of Technology, Atlanta, GA 30394, USA; 3 Department of Communication Science and Disorders, School of Health and Rehabilitation Sciences, University of Pittsburgh, Pittsburgh, PA 15260, USA

**Keywords:** electrocardiogram, emergency department, machine learning, natural language processing, clinical decision support

## Abstract

**Background**: Electrocardiograms (EKGs) are essential tools in emergency medicine, often used to evaluate chest pain, dyspnea, and other symptoms suggestive of cardiac dysfunction. Yet, EKGs are not universally administered to all emergency department (ED) patients. Understanding and predicting which patients receive an EKG may offer insights into clinical decision making, resource allocation, and potential disparities in care. This study examines whether integrating structured clinical data with free-text patient narratives can improve prediction of EKG utilization in the ED. **Methods**: We conducted a retrospective observational study to predict electrocardiogram (EKG) utilization using data from 13,115 adult emergency department (ED) visits in the nationally representative 2021 National Hospital Ambulatory Medical Care Survey–Emergency Department (NHAMCS-ED), leveraging both structured features—demographics, vital signs, comorbidities, arrival mode, and triage acuity, with the most influential selected via Lasso regression—and unstructured patient narratives transformed into numerical embeddings using Clinical-BERT. Four supervised learning models—Logistic Regression (LR), Support Vector Machine (SVM), Random Forest (RF) and Extreme Gradient Boosting (XGB)—were trained on three inputs (structured data only, text embeddings only, and a late-fusion combined model); hyperparameters were optimized by grid search with 5-fold cross-validation; performance was evaluated via AUROC, accuracy, sensitivity, specificity and precision; and interpretability was assessed using SHAP values and Permutation Feature Importance. **Results**: EKGs were administered in 30.6% of adult ED visits. Patients who received EKGs were more likely to be older, White, Medicare-insured, and to present with abnormal vital signs or higher triage severity. Across all models, the combined data approach yielded superior predictive performance. The SVM and LR achieved the highest area under the ROC curve (AUC = 0.860 and 0.861) when using both structured and unstructured data, compared to 0.772 with structured data alone and 0.823 and 0.822 with unstructured data alone. Similar improvements were observed in accuracy, sensitivity, and specificity. **Conclusions**: Integrating structured clinical data with patient narratives significantly enhances the ability to predict EKG utilization in the emergency department. These findings support a personalized medicine framework by demonstrating how multimodal data integration can enable individualized, real-time decision support in the ED.

## 1. Introduction

Electrocardiograms (EKGs) are a cornerstone of cardiovascular evaluation in emergency medicine [1,2]. They provide rapid, non-invasive insights into cardiac function and are essential in diagnosing life-threatening conditions such as myocardial infarction, arrhythmias, and pulmonary embolism. Despite their clinical importance, EKGs are not ordered for every emergency department (ED) patient. Rather, decisions about EKG utilization are often made under conditions of time pressure and diagnostic uncertainty, guided by a complex interplay of presenting symptoms, vital signs, comorbidities, and clinical judgment [3,4]. The integration of multimodal data to predict diagnostic testing exemplifies a personalized medicine approach, where models consider not only population-level trends but also individual-level contextual information.

Understanding which patients are more likely to receive an EKG—and why—has implications beyond clinical efficiency. Patterns in EKG utilization may reflect differences in disease severity, access to care, implicit biases, or institutional protocols [5,6]. More importantly, such patterns may signal potential gaps in care delivery, especially among populations who may present with atypical symptoms or have less clearly defined risk profiles. Yet, to date, few studies have systematically explored the factors driving EKG use across a large, nationally representative cohort, and fewer still have attempted to predict EKG utilization using a multimodal data framework.

Recent advances in machine learning and natural language processing (NLP) have opened new possibilities for leveraging both structured and unstructured clinical data to support clinical decision making [7,8,9,10,11]. Structured electronic health record (EHR) data—such as demographics, vital signs, and coded comorbidities—offer measurable predictors of care patterns [12,13]. However, unstructured text, such as patient-reported chief complaints and narrative triage notes, often carries critical clinical nuance that is lost in structured fields [14,15]. By embedding these narratives using transformer-based models like Clinical-BERT [16,17], we can capture subtle semantic cues from patient descriptions and transform them into features suitable for machine learning models [18,19].

In this study, we sought to predict EKG utilization in the emergency department by integrating structured clinical data with embedded representations of patient narratives. Using data from the 2021 National Hospital Ambulatory Medical Care Survey–Emergency Department (NHAMCS-ED), we trained and evaluated four machine learning models—Logistic Regression, Support Vector Machine, Random Forest, and gradient boosting classifier—across three data configurations: structured only, unstructured only, and combined. Our work advances the goals of personalized medicine by enabling patient-specific predictions of EKG utilization based on comprehensive clinical information. By analyzing both vital signs and semantically rich narratives, our models can reflect the complexity and individuality of real-world presentations. This level of personalization is critical in acute care settings, where rapid, nuanced decisions are essential. The methods demonstrated here are scalable to other diagnostic decisions and have potential to improve care delivery by reducing one-size-fits-all approaches.

## 2. Methods

### 2.1. Study Design and Data Source

We conducted a retrospective observational study using data from the 2021 NHAMCS-ED [20,21]. The NHAMCS-ED is a nationally representative survey conducted annually by the National Center for Health Statistics (NCHS), part of the U.S. Centers for Disease Control and Prevention (CDC). Designed to characterize patterns of emergency care across the United States, NHAMCS-ED employs a multistage probability sampling strategy that captures visits from non-institutional, non-federal, general, and short-stay hospitals. The dataset includes detailed information on patient demographics, visit characteristics, triage acuity, vital signs, diagnostic tests, procedures, and medications, along with free-text narratives describing the patient’s chief complaint and reason for visit. The 2021 dataset, used in this study, is particularly valuable for research on healthcare utilization because it reflects post-pandemic ED visit patterns and integrates both structured and unstructured data elements. Its standardized data collection methodology allows for robust national estimates, making it an essential resource for epidemiologic and health services research focused on emergency care delivery.

Our analytic sample included 13,115 adult visits from individuals aged 18 years or older. We excluded pediatric cases and records with missing or incomplete key variables related to the outcome or main predictors. The primary outcome was whether an EKG was performed during the ED visit.

### 2.2. Variable Selection and Data Processing

We used both structured and unstructured data elements to model the probability of EKG utilization.

Structured data encompassed patient demographics (age, sex, race/ethnicity), visit characteristics (arrival time, mode of arrival, visit type, whether the visit was an initial or follow-up encounter), and vital signs (temperature, heart rate, respiratory rate, blood pressure, oxygen saturation, and pain score). Clinical severity was captured using the Emergency Severity Index (ESI) triage scale, and we also extracted indicators of comorbid conditions such as diabetes, hypertension, congestive heart failure, coronary artery disease, chronic kidney disease, and others. Additional variables included insurance type, residence status (e.g., private home, nursing facility, or homeless), and whether the visit involved trauma, overdose, or adverse effects from medical care.

Unstructured data included free-text entries describing the patient’s chief complaint and any reported reason for injury or illness. These narratives were preprocessed and embedded using Clinical-BERT, a deep learning model that converts clinical text into semantically meaningful vector representations while preserving contextual nuance.

To address missing values in structured fields, we applied median imputation, which allowed us to retain the full cohort for analysis without introducing bias through listwise deletion.

We also used multivariable Logistic Regression to examine the association between structured patient characteristics and EKG utilization. Adjusted odds ratios and 95% confidence intervals were estimated, and the results were visualized using a forest plot to highlight the most influential factors associated with EKG use during the ED visit.

Because the NHAMCS-ED dataset is cross-sectional and visit-based rather than patient-based, individual patients cannot be tracked across encounters; therefore, each emergency department visit is treated as an independent observation, and repeated visits by the same individual cannot be identified.

### 2.3. Feature Selection and Predictive Modeling

To reduce dimensionality and enhance the interpretability of structured variables, we applied Lasso regression (L1 penalty) on the full set of structured predictors. Features with non-zero coefficients comprised the final parsimonious set used for model building.

To evaluate the predictive capacity for EKG utilization in the emergency department, we implemented four supervised machine learning algorithms: Logistic Regression (LR), Support Vector Machine (SVM), Random Forest (RF), and Extreme Gradient Boosting (XGB). These models were selected to represent a spectrum of linear and non-linear classifiers, allowing for comprehensive performance comparison across different functional forms.

For Logistic Regression, we employed an L2 regularization penalty with class_weight = ‘balanced’ to account for the moderate class imbalance. A grid search was conducted over a range of regularization strengths, C = {0.01, 0.1, 1, 10}, using the lbfgs solver and a maximum of 2000 iterations to ensure convergence.

The SVM model utilized a linear kernel and class_weight = ‘balanced’, with hyperparameter tuning over C = {0.1, 1, 10}. The dual = False option was applied to improve efficiency for datasets with more observations than features, as was the case in our structured inputs.

The Random Forest classifier was configured with a grid search over key hyperparameters, including the number of trees (n_estimators = {100, 200}), tree depth (max_depth = {None, 10}), and minimum number of samples required to split an internal node (min_samples_split = {2, 5}). This ensemble method provided a non-parametric benchmark capable of capturing complex interactions among features.

For XGBoost, a gradient boosting method known for its scalability and predictive performance, we explored combinations of n_estimators = {100, 200}, learning_rate = {0.1, 0.2}, max_depth = {3, 5}, and subsample = {0.8, 1.0}. These parameters control the learning dynamics and regularization, crucial for preventing overfitting in boosted tree models.

Each model was trained under three input conditions to examine the contribution of different data modalities: (1) structured data alone, encompassing demographics, vital signs, comorbidities, and triage characteristics; (2) unstructured data alone, consisting of narrative chief complaints embedded using Clinical-BERT; and (3) a multimodal configuration combining both structured and unstructured features. This design enabled us to systematically evaluate the additive value of patient narratives in predictive modeling. Hyperparameters were optimized via grid search with 5-fold cross-validation to ensure robust generalization. The late-fusion model averaged predicted probabilities from the best-performing structured and text-only models.

### 2.4. Model Evaluation

To judge performance, we calculated standard classification metrics, including accuracy, precision, sensitivity, specificity, and the area under the receiver operating characteristic curve (AUC-ROC). ROC curves were plotted for visual comparison, and optimal decision thresholds were selected by balancing sensitivity and specificity, reflecting the real-world tradeoffs clinicians face in emergency settings.

To identify the factors driving model predictions, we applied two complementary interpretability techniques. Permutation Feature Importance was used to assess the global influence of each predictor by quantifying the decrease in AUC-ROC when the values of a given feature were randomly permuted. This method allowed us to determine which variables contributed most to the overall discriminative performance of each model.

In addition, we employed SHAP (SHapley Additive exPlanations) values to provide both global and local interpretability. SHAP values quantify the marginal contribution of each feature to individual predictions while summarizing feature effects across the entire cohort. This approach enabled us to capture non-linear interactions between structured and unstructured features, offering a more nuanced understanding of model behavior.

By leveraging these interpretability methods within our multimodal framework, we sought not only to improve predictive accuracy for EKG utilization but also to gain clinical insights into how structured variables and patient narratives jointly influence decision-making processes in acute care settings.

This research utilized publicly available, anonymized data from NHAMCS-ED, managed by the U.S. Centers for Disease Control and Prevention. As this secondary analysis involved de-identified data, the study received exempt approval from the Institutional Review Board (IRB) at the University of Pittsburgh (protocol STUDY24120115), adhering to ethical guidelines for secondary data use.

## 3. Results

**Figure 1:**
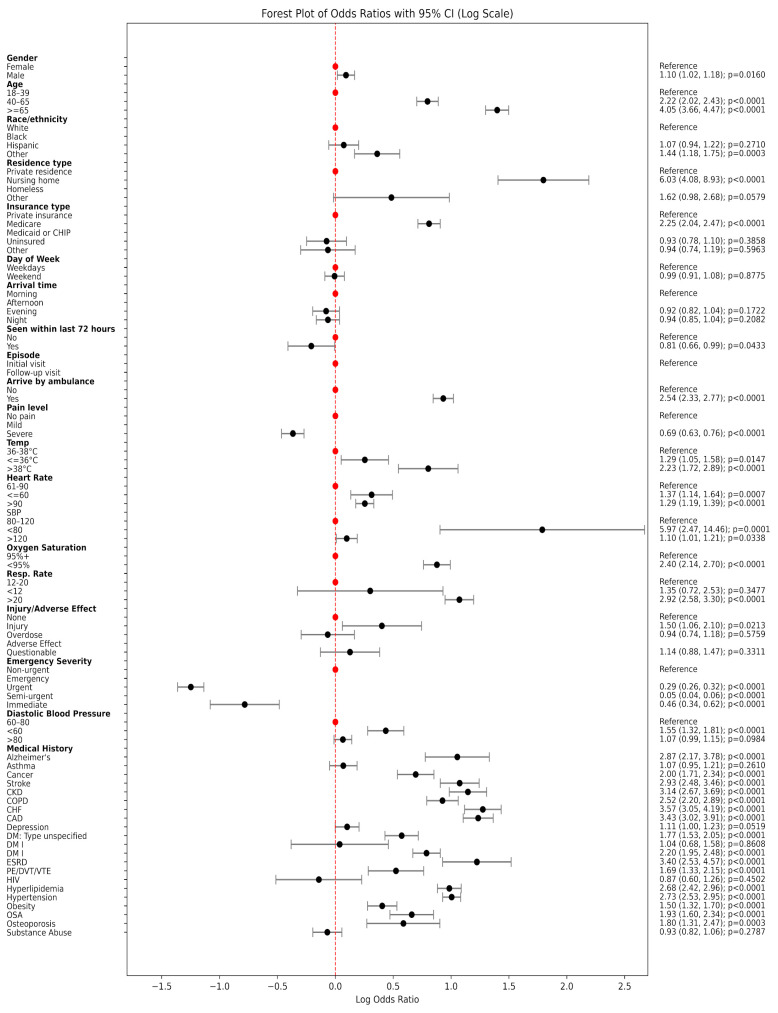
Forest plot of odds ratios with 95% CI (Log Scale).

We analyzed data from 13,115 adult ED visits, of which 4010 patients (30.6%) received an EKG during their visit. Patients who received an EKG differed substantially from those who did not across a wide range of demographic, clinical, and contextual characteristics (Table 1 and Figure 1).

### 3.1. Patient Characteristics Associated with EKG Utilization

Patients who underwent EKG testing were notably older: over a third (37.0%) were aged 65 years or older, compared to only 18.1% in the non-EKG group (*p* < 0.0001). They were also more likely to be White (61.4% vs. 57.9%, *p* < 0.0001) and less likely to be Black, Hispanic, homeless, or uninsured. The EKG group had a higher proportion of nursing home residents (3.4% vs. 1.4%, *p* < 0.0001) and Medicare beneficiaries (44.7% vs. 29.1%, *p* < 0.0001), suggesting both older age and chronic illness influenced testing decisions.

Clinically, patients who received EKGs were more likely to arrive by ambulance (31.9% vs. 15.6%, *p* < 0.0001) and to present with more severe physiological signs. Elevated body temperature, tachycardia (heart rate > 90 bpm), tachypnea (respiratory rate > 20 breaths/min), and hypoxemia (SpO_2_ ≤ 94%) were all significantly more common in the EKG group (all *p* < 0.0001). In terms of triage acuity, 24.6% of patients receiving an EKG were categorized as emergency or immediate based on the Emergency Severity Index (ESI), compared to just 7.7% in those who did not receive an EKG.

Chronic health conditions were also more prevalent among EKG recipients. Rates of congestive heart failure (10.0% vs. 3.0%), coronary artery disease (14.8% vs. 4.8%), chronic kidney disease (9.0% vs. 3.0%), type II diabetes (14.5% vs. 7.2%), and hypertension (48.7% vs. 25.8%) were all significantly higher among those who received an EKG (*p* < 0.0001). These differences underscore the influence of cardiovascular and metabolic comorbidities in clinical decision making around EKG use.

### 3.2. Predictive Models

The feature selection process substantially reduced the overall dimensionality of the dataset from 809 to 258 total features, creating a more parsimonious and computationally efficient set of predictors. For the structured data, Lasso regression narrowed the initial 41 variables down to 33, retaining key predictors such as patient demographics (age, sex, race/ethnicity), vital signs (heart rate, systolic blood pressure), and relevant comorbidities like coronary artery disease and hypertension. Concurrently, the dimensionality of the unstructured text embeddings was reduced from 768 to 225. This significant reduction helps to mitigate the risk of overfitting while ensuring the final models are trained on the most informative variables.

**Figure 2:**
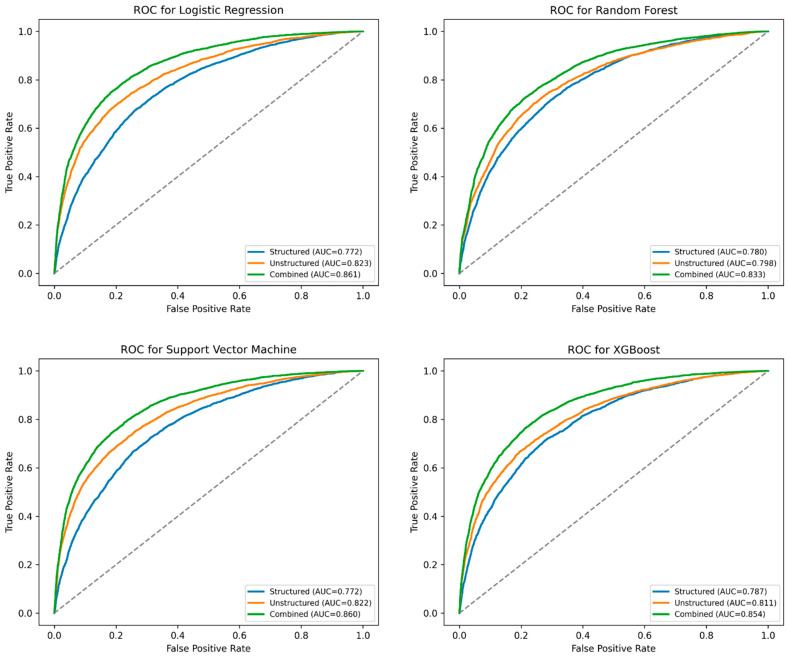
Mean ROC curve for Logistic Regression (LR), Support Vector Machine (SVM), Random Forest (RF), and XG Boost (XGB) models predicting EKG Use.

Figure 2 and Table 2 summarize the performance of four machine learning models—Logistic Regression, Support Vector Machine (SVM), Random Forest, and XG Boost (XGB)—trained to predict EKG utilization using three different types of input data: structured data only, unstructured patient narratives only, and a combination of both.

Across all models, the combined approach consistently outperformed models that relied solely on structured or unstructured data. For instance, in the Logistic Regression model, the area under the ROC curve (AUC) improved from 0.772 (structured-only) and 0.823 (unstructured-only) to 0.861 when the two data types were integrated. A similar pattern was observed in the SVM model, where the AUC increased from 0.772 (structured) and 0.822 (unstructured) to 0.859 with combined data.

Accuracy, sensitivity, and specificity metrics followed similar trends. The combined SVM model achieved an overall accuracy of 77.7%, with a sensitivity of 78.6% and specificity of 77.4%, indicating a strong balance in correctly identifying both EKG and non-EKG cases. The combined XGB and Random Forest models also performed well, with AUCs of 0.854 and 0.833, respectively.

### 3.3. Model Interpretability

**Figure 3:**
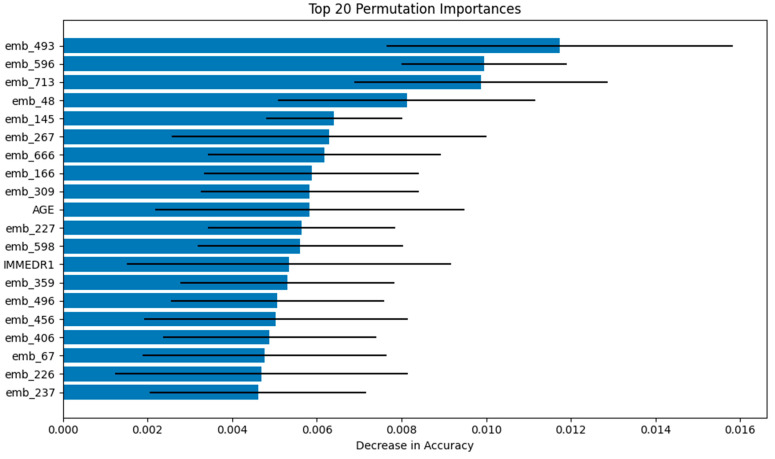
Visualizations of Permutation Feature Importance for Logistic Regression (Top 20 features).

The interpretability of the Logistic Regression model was evaluated using Permutation Feature Importance, which measures the reduction in model performance (AU-ROC) when individual feature values are randomly permuted. This analysis demonstrated that the text embeddings derived from patient narratives were the most influential predictors within the LR model. Notably, the top three embedding features—emb_493, emb_596, and emb_713—resulted in the greatest decrease in predictive accuracy when permuted. Although the embeddings dominated feature importance, structured clinical variables such as age (AGE) and immediate reason for visit (IMMEDR1) also ranked among the top 20 most impactful predictors. These findings indicate that the LR model achieved its predictive performance through a synergistic integration of both narrative-derived and structured features (Figure 3).

**Figure 4:**
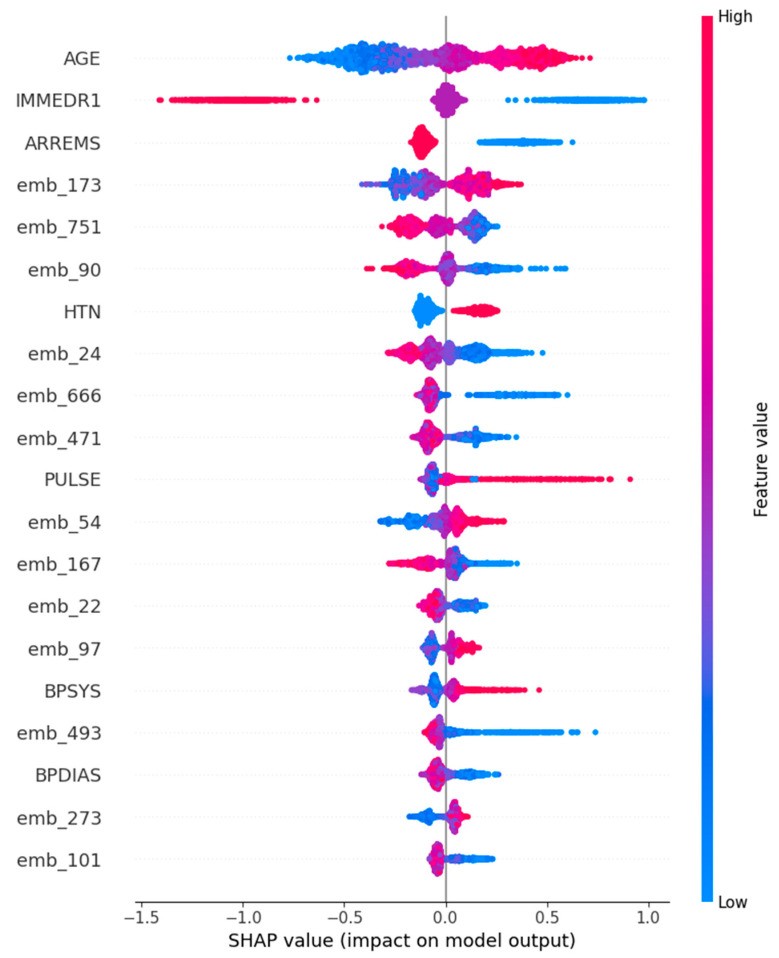
Visualizations of SHapley Additive exPlanations for Support Vector Machine (Top 20 features).

For the Support Vector Machine model, interpretability was examined using SHAP values, which provide both local and global insights into feature contributions. The SHAP summary plot revealed that age was the most influential feature overall, with higher age values consistently associated with positive SHAP values, thereby increasing the probability of predicting EKG utilization. Other structured variables, such as arrival by emergency medical services (ARREMSs) and elevated pulse (PULSE), also exhibited strong positive associations with EKG predictions. Moreover, SHAP analysis uncovered the non-linear contributions of text embeddings (e.g., emb_173 and emb_90), illustrating how the SVM model capitalized on the nuanced semantic information present in patient chief complaints to refine its predictions (Figure 4).

## 4. Discussions

This study demonstrates that integrating structured clinical variables with unstructured patient narratives significantly enhances the prediction of EKG utilization in emergency departments. Across all four machine learning models evaluated, the combination of structured features (e.g., demographics, vital signs, comorbidities) with Clinical-BERT text embeddings yielded the highest predictive performance, with Logistic Regression and SVM achieving AUCs exceeding 0.85. These findings highlight the potential of multimodal approaches to capture the complexity of clinical decision making, complementing recent efforts to leverage artificial intelligence for optimizing ECG testing and interpretation [1,2].

Our results align with prior studies that emphasize the diagnostic value of unstructured narratives. For example, Zhang et al. [10,12] and Chang et al. [7] reported that incorporating free-text triage data can significantly improve predictions of ED disposition and hospital admission. Similarly, Stewart et al. [8] reviewed the advantages of NLP in triage, emphasizing that patient narratives often encode subtle clinical cues—such as chest discomfort or intermittent shortness of breath—that structured variables alone may miss. The improved performance of our models reflects the ability of Clinical-BERT to capture these nuances, providing richer feature representations than traditional NLP techniques [16,17,18].

We observed that demographic and insurance-related factors were associated with EKG utilization, with higher testing rates among older, White, and Medicare-insured patients. These findings are consistent with previous studies documenting disparities in cardiovascular diagnostic testing [5,6]. While race and insurance status are not clinical risk factors, they were included in our models for two purposes: (1) to identify and quantify potential inequities in current ED practice, and (2) to adjust for confounding influences that could otherwise bias the associations between clinical predictors and EKG utilization. This approach is aligned with health disparities research, which seeks to expose, rather than perpetuate, systemic inequities. Importantly, we emphasize that these variables would require fairness audits and mitigation strategies before any clinical deployment of our models.

Our interpretability analyses further revealed that both structured and narrative data contributed meaningfully to prediction. Text embeddings derived from patient chief complaints were the dominant predictors in the Logistic Regression model, while SHAP analysis of the SVM model highlighted age, arrival by EMS, and elevated heart rate as key structured factors influencing predictions. These findings are clinically intuitive, as older patients and those arriving via EMS typically present with higher acuity and cardiovascular risk.

Despite these strengths, several limitations merit consideration. First, the NHAMCS-ED dataset is cross-sectional and visit-based, preventing identification of repeated visits by the same individual or longitudinal outcomes. Second, our models predict the utilization of EKGs, not their appropriateness or clinical outcomes (e.g., myocardial infarction diagnosis). As Sanjay et al. [4] noted, the value of EKG testing depends on patient risk stratification, and future work should link predictive models to downstream clinical outcomes to evaluate impact. Third, while we filtered text data for terms explicitly referencing EKG orders or results, residual data leakage cannot be entirely excluded, although performance metrics remained stable after these terms were removed. Lastly, our analysis is based on 2021 data, a period still affected by the COVID-19 pandemic, which has been shown to alter ED utilization patterns [22].

Future research should validate these findings with post-pandemic data and explore fairness-aware AI frameworks to ensure equitable recommendations. Integrating outcome-linked data (e.g., cardiovascular diagnoses or adverse events) and assessing cost-effectiveness and workflow integration would also be important next steps for translating this work into clinical decision support.

## 5. Conclusions

By integrating structured data with patient narratives, this study advances a more nuanced and accurate approach to predicting EKG utilization in the ED. The findings illustrate the untapped potential of unstructured clinical text and affirm the value of combining traditional statistical predictors with modern NLP techniques. As emergency departments continue to grapple with diagnostic complexity and workflow strain, tools that intelligently synthesize diverse data sources may help ensure timely and equitable care for all patients. Future applications of this multimodal prediction framework could extend beyond EKG use to other diagnostic and therapeutic decisions, further supporting the goals of personalized medicine in emergency care settings.

## Figures and Tables

**Table 1:** Demographic and clinical characteristics of emergency department patients categorized by EKG Use.

	EKG Use		
	No	Yes	*p* Value
	9105 (69.4%)	4010 (30.6%)	
Gender			0.0168
Female	4980 (54.7%)	2102 (52.4%)	
Male	4125 (45.3%)	1908 (47.6%)	
Age			<0.0001
18–39	4222 (46.4%)	937 (23.4%)	
40–65	3233 (35.5%)	1591 (39.7%)	
> 65	1650 (18.1%)	1482 (37.0%)	
Race/ethnicity			<0.0001
White	5273 (57.9%)	2464 (61.4%)	
Black	2192 (24.1%)	831 (20.7%)	
Hispanic	1298 (14.3%)	529 (13.2%)	
Other	342 (3.8%)	186 (4.6%)	
Residence type			<0.0001
Private residence	8597 (94.4%)	3794 (94.6%)	
Nursing home	129 (1.4%)	137 (3.4%)	
Homeless	267 (2.9%)	47 (1.2%)	
Other	112 (1.2%)	32 (0.8%)	
Insurance type			<0.0001
Private insurance	2445 (26.9%)	1037 (25.9%)	
Medicare	2652 (29.1%)	1791 (44.7%)	
Medicaid or CHIP	2921 (32.1%)	878 (21.9%)	
Uninsured	736 (8.1%)	205 (5.1%)	
Other	351 (3.9%)	99 (2.5%)	
Day of Week			0.8945
Weekdays	6691 (73.5%)	2952 (73.6%)	
Weekend	2414 (26.5%)	1058 (26.4%)	
Arrival time			0.3963
Morning	2544 (27.9%)	1143 (28.5%)	
Afternoon	2956 (32.5%)	1342 (33.5%)	
Evening	1418 (15.6%)	594 (14.8%)	
Night	2187 (24.0%)	931 (23.2%)	
Seen within last 72 h			0.0483
No	8736 (95.9%)	3877 (96.7%)	
Yes	369 (4.1%)	133 (3.3%)	
Episode			<0.0001
Initial visit	8400 (92.3%)	3803 (94.8%)	
Follow-up visit	705 (7.7%)	207 (5.2%)	
Arrive by ambulance			<0.0001
No	7689 (84.4%)	2732 (68.1%)	
Yes	1416 (15.6%)	1278 (31.9%)	
Pain level			<0.0001
No pain	1933 (21.2%)	1191 (29.7%)	
Mild	4758 (52.3%)	2086 (52.0%)	
Severe	2414 (26.5%)	733 (18.3%)	
Temperature			<0.0001
36–38 °C	8717 (95.7%)	3746 (93.4%)	
< 36 °C	267 (2.9%)	148 (3.7%)	
>38 °C	121 (1.3%)	116 (2.9%)	
Heart Rate			<0.0001
61–90	5544 (60.9%)	2187 (54.5%)	
< 60	365 (4.0%)	197 (4.9%)	
>90	3196 (35.1%)	1626 (40.5%)	
SBP			<0.0001
80–120	2064 (22.7%)	839 (20.9%)	
<80	7 (0.1%)	17 (0.4%)	
>120	7034 (77.3%)	3154 (78.7%)	
DBP			<0.0001
60–80	4326 (47.5%)	1799 (44.9%)	
<60	459 (5.0%)	295 (7.4%)	
>80	4319 (47.4%)	1916 (47.8%)	
Oxygen Saturation			<0.0001
95%+	8443 (92.7%)	3374 (84.1%)	
<95%	662 (7.3%)	636 (15.9%)	
Resp. Rate			<0.0001
12–20	8556 (94.0%)	3392 (84.6%)	
<12	28 (0.3%)	15 (0.4%)	
>20	521 (5.7%)	603 (15.0%)	
Injury/Adverse Effect			
None	2661 (29.2%)	491 (12.2%)	
Injury	84 (0.9%)	55 (1.4%)	
Overdose	256 (2.8%)	105 (2.6%)	
Adverse Effect	5923 (65.1%)	3269 (81.5%)	
Questionable	181 (2.0%)	90 (2.2%)	
Emergency Severity			<0.0001
Immediate	118 (1.3%)	84 (2.1%)	
Emergency	579 (6.4%)	902 (22.5%)	
Urgent	6374 (70.0%)	2847 (71.0%)	
Semi-urgent	1793 (19.7%)	146 (3.6%)	
Non-urgent	241 (2.6%)	31 (0.8%)	
Medical History			
Alzheimer’s disease/Dementia	92 (1.0%)	114 (2.8%)	<0.0001
Asthma	955 (10.5%)	447 (11.1%)	0.2741
Cancer	356 (3.9%)	302 (7.5%)	<0.0001
Cerebrovascular disease/History of stroke (CVA)	262 (2.9%)	320 (8.0%)	<0.0001
Chronic kidney disease (CKD)	277 (3.0%)	360 (9.0%)	<0.0001
Chronic obstructive pulmonary disease (COPD)	450 (4.9%)	465 (11.6%)	<0.0001
Congestive heart failure (CHF)	276 (3.0%)	403 (10.0%)	<0.0001
Coronary artery disease (CAD)	438 (4.8%)	593 (14.8%)	<0.0001
Depression	1331 (14.6%)	639 (15.9%)	0.0551
Diabetes mellitus (DM)-Type unspecified	466 (5.1%)	350 (8.7%)	<0.0001
Diabetes mellitus (DM))-Type I	70 (0.8%)	32 (0.8%)	0.9462
Diabetes mellitus (DM)-Type II	654 (7.2%)	583 (14.5%)	<0.0001
End-stage renal disease (ESRD)	75 (0.8%)	110 (2.7%)	<0.0001
Pulmonary embolism (PE), DVT, or venous thromboembolism (VTE)	162 (1.8%)	119 (3.0%)	<0.0001
HIV infection/AIDS	102 (1.1%)	39 (1.0%)	0.5068
Hyperlipidemia	869 (9.5%)	883 (22.0%)	<0.0001
Hypertension	2347 (25.8%)	1952 (48.7%)	<0.0001
Obesity (BMI ≥ 30)	674 (7.4%)	429 (10.7%)	<0.0001
Obstructive sleep apnea (OSA)	248 (2.7%)	206 (5.1%)	<0.0001
Osteoporosis	89 (1.0%)	70 (1.7%)	0.0003
Substance abuse or dependence	928 (10.2%)	384 (9.6%)	0.2929

Note: The variables “Respiratory Rate,” “Temperature,” “Pulse Oximetry,” “Heart Rate,” “Payment Type,” “Seen Within Last 72 Hours,” and “Episode of Care” have missing data proportions ranging between 5% and 10%. The variables “Arrival Time,” “Patient Residence,” “Arrival by Ambulance,” “Systolic Blood Pressure,” “Diastolic Blood Pressure,” and “Visit Related to Injury/Trauma, Overdose/Poisoning, or Adverse Effect of Medical/Surgical Treatment” have missing data proportions of less than 5%.

**Table 2:** Prediction performance comparison for Logistic Regression (LR), Support Vector Machine (SVM), Random Forest (RF), and XG Boost (XGB).

Model	Feature Set	Accuracy	Precision	Sensitivity	Specificity	AUC	Best Parameter
Logistic Regression (LR)	Combined	0.794	0.637	0.755	0.811	0.861	{‘C’: 0.1, ‘penalty’: ‘l2’, ‘solver’: ‘lbfgs’}
	Unstructured	0.766	0.600	0.703	0.793	0.823	{‘C’: 0.1, ‘penalty’: ‘l2’, ‘solver’: ‘lbfgs’}
	Structured	0.717	0.530	0.679	0.734	0.772	{‘C’: 0.01, ‘penalty’: ‘l2’, ‘solver’: ‘lbfgs’}
Random Forest (RF)	Combined	0.770	0.604	0.722	0.792	0.833	{‘max_depth’: 10, ‘min_samples_split’: 2, ‘n_estimators’: 200}
	Unstructured	0.742	0.563	0.700	0.761	0.798	{‘max_depth’: 10, ‘min_samples_split’: 5, ‘n_estimators’: 200}
	Structured	0.709	0.517	0.715	0.706	0.780	{‘max_depth’: 10, ‘min_samples_split’: 5, ‘n_estimators’: 200}
Support Vector Machine (SVM)	Combined	0.777	0.605	0.786	0.774	0.860	{‘C’: 0.1}
	Unstructured	0.751	0.572	0.730	0.760	0.822	{‘C’: 0.1}
	Structured	0.699	0.506	0.725	0.688	0.772	{‘C’: 0.1}
XG Boosting (XGB)	Combined	0.778	0.609	0.768	0.783	0.854	{‘learning_rate’: 0.1, ‘max_depth’: 3, ‘n_estimators’: 200, ‘subsample’: 1.0}
	Unstructured	0.721	0.532	0.711	0.725	0.787	{‘learning_rate’: 0.1, ‘max_depth’: 3, ‘n_estimators’: 100, ‘subsample’: 0.8}
	Structured	0.763	0.601	0.667	0.805	0.811	{‘learning_rate’: 0.1, ‘max_depth’: 3, ‘n_estimators’: 200, ‘subsample’: 0.8}

## Data Availability

The NHAMCS-ED dataset can be accessed through the website of the US Centers for Disease Control and Prevention (CDC) (https://www.cdc.gov/nchs/namcs/about/?CDC_AAref_Val=, accessed on 1 January 2025). The detailed explanation of the survey data for each year and the code book can be found here: https://ftp.cdc.gov/pub/Health_Statistics/NCHS/dataset_documentation/nhamcs/, accessed on 1 January 2025. The SAS dataset for each year can be found here: https://ftp.cdc.gov/pub/Health_Statistics/NCHS/Datasets/NHAMCS/, accessed on 1 January 2025. All the data were in a SAS format. To obtain the unstructured data, one needs to run the SAS format files under the following link before import the data to the analysis software. https://ftp.cdc.gov/pub/Health_Statistics/NCHS/dataset_documentation/nhamcs/sas/, accessed on 1 January 2025.
